# 28S rRNA sequences for *Linguatula* spp.

**DOI:** 10.1007/s00436-022-07507-6

**Published:** 2022-04-01

**Authors:** Shokoofeh Shamsi, Xiaocheng Zhu, Ali Halajian, Diane P. Barton

**Affiliations:** 1 School of Agricultural, Environmental and Veterinary Sciences, Charles Sturt University, Wagga Wagga, NSW 2678 Australia; 2 Wagga Wagga Agricultural Institute, New South Wales Department of Primary Industries, Wagga Wagga, NSW 2678 Australia; 3 DSI-NRF SARChI Chair (Ecosystem Health), Department of Biodiversity, University of Limpopo, Sovenga, South Africa; 4 Research Administration and Development, University of Limpopo, Sovenga, South Africa

**Keywords:** Pentastomida, Molecular sequences, Phylogeny, *Linguatulaserrata*, *Linguatulanuttalli*

## Abstract

Identification of specimens belonging to the genus *Linguatula* (Pentastomida) is relatively easy due to their unique morphology. However, differentiation between species of *Linguatula* can be challenging for several reasons, including considerable differences between different developmental stages of the parasite within and between species. Currently, 18S rRNA and *Cox1* sequences are the only available comparable sequences in GenBank, but recent research has discussed the utility of 28S rRNA for pentastomid phylogenetics. This study presents 28S rRNA gene sequences for two members of the genus *Linguatula*. Sequences of 28S rRNA were successfully obtained from well-identified samples of *L.* *serrata* (collected in Australia) and *L.* *nuttalli* (collected in South Africa), with voucher specimens. Phylogenetic analysis of the 28S rRNA region showed 6% difference between *L.* *serrata* and *L.* *nuttalli*, with low levels of intraspecific variation. In comparison, 18S rRNA and *Cox1* sequences from the same specimens showed 0.23% and 13% interspecific differences, respectively. The results of this study show that 28S rRNA has greater genetic diversity to allow for improved differentiation between species of *Linguatula* than 18S rRNA but is on par with *Cox1*. Records that do not provide adequate morphological or molecular data to justify independent specific diagnoses must be regarded cautiously, and the need for continued research on species of *Linguatula*, using a combined morphological and molecular analysis, across a number of different hosts, development stages, geographical regions and molecular markers is highlighted.

**Supplementary Information:**

The online version contains supplementary material available at 10.1007/s00436-022-07507-6.

## Introduction

*Linguatula* spp., belonging to the Pentastomida, are obligatory arthropod parasites which have an indirect life cycle. When adult, they inhabit the nasal cavity of their definitive hosts, which usually is a carnivorous mammal, such as a canid (Shamsi et al. 2017b) or a felid (Shamsi et al. 2020b). They produce eggs which are expelled to the environment through faeces or nasal discharge. When ingested by the intermediate host, usually a herbivorous mammal (Barton et al. 2020a, 2020b), the eggs hatch and the parasite migrates through various organs such as the lung, liver, and lymph nodes (Basson et al. 1970) where the nymph undergoes development. The parasite life cycle is completed when an infected intermediate host is ingested by the definitive host. *Linguatula* spp. are known to be pathogenic for both definitive and intermediate hosts (Godara et al. 2013; Shamsi et al. 2018). They are also commonly reported from humans (Tabaripour et al. 2021). Despite their veterinary and medical significance, the taxonomy and classification of these parasites have been confusing and often contradictory (Christoffersen and de Assis 2013; Poore 2012) which, along with the worldwide shortage of taxonomists, has resulted in difficulties in specifically and accurately identifying these parasites.

At present, the genus is comprised of five valid species (Christoffersen and de Assis 2013; Poore 2012): *L.* *arctica* Riley et al., 1987; *L.* *multiannulata* Haffner, Sachs & Rack, 1967; *L.* *nuttalli* Sambon, 1922; *L.* *recurvata* (Diesing, 1850); and *L.* *serrata* Frölich, 1789. Although identification to genus is relatively easy, differentiation between species of *Linguatula* can be challenging for a number of reasons. Morphologically, there are considerable differences between different developmental stages of the parasite within and between species. The taxonomic value of these differences is not yet fully understood. As a result, it is not surprising that the taxonomy and classification of these parasites have been problematic (Christoffersen and de Assis 2013; Poore 2012).

Of the reported species, *L.* *serrata* seems to be the most widespread and the most studied *Linguatula*. It is believed that *L.* *serrata* has been spread from Europe to other continents through human movements involving movement of infected dogs and cattle (Ortlepp 1934; Shamsi et al. 2020a). However, there are many publications that have based their identification of the parasite on the assumption that any pentastome removed from the nasal cavity of a mammal is *L.* *serrata*. Indeed, pentastomes collected from the nasal passages of reindeer were initially reported as *L.* *serrata* by Chapin (1926) and others, until differences in morphology and type of definitive host and a putative direct life cycle eventually led to its description as a new species, *L.* *arctica*, by Riley et al. (1987). Additionally, many reports of *L.* *serrata* are based on the observation of nymphal specimens which, like the adult, are automatically assumed to be *L.* *serrata* without corresponding morphological characterisation (see Pérez-Flores et al. 2019).

Recently, genetic identification has been undertaken, but quite often without providing sufficient justification for species identification (e.g., Ghorashi et al. 2016; Naude et al. 2018; Sudan et al. 2018; Mohammadi et al. 2020), leading to the potential of mis-identified genetic sequences to further confuse and compromise future studies. The absence of well identified/described specimens, with representative vouchered museum specimens, prevents the sequences from truly clarifying or verifying taxonomic identifications. This has added to the current poor understanding of the fundamental aspects of these parasites and the ability to accurately diagnose infections.

For example, in a study on pentastomid nymphs collected from herbivores in Iran, partial sequences of 18S rRNA were used to assign them to *L.* *serrata* (Ghorashi et al. 2016). In a latter study (Shamsi et al. 2020b), sequences of these pentastomid nymphs formed a group distinct from *L.* *serrata* reported in Europe, suggesting that they belong to a different, as yet unknown, species. Although adult *Linguatula* have been collected from dogs in Iran, there has been no morphological description of adult *Linguatula* in the country to confirm the specific identity and taxonomic status of the parasite in Iran. Indeed, the sequence obtained by Ghorashi et al. (2016) for an adult specimen of *Linguatula* collected from an Iranian dog did not match with the sequence provided by Gjerde (2013).

Currently, 18S rRNA and *Cox1* sequences are the only available comparable sequences in GenBank. Shamsi et al. (2020b), in their work on *L.* *nuttalli,* stated that as these two regions are two independent gene targets, they provide independent views of the phylogenetic relationships among species. However, low levels of genetic variability in 18S rRNA sequences, as found for *Linguatula* spp. (Gjerde 2013; Shamsi et al. 2020a, b), make it difficult to differentiate species level identifications (Literák et al. 2017). In a recent work on *Levisunguis* *subaequalis* (Pentastomida), Woodyard et al. (2019a) discussed the utility of the 28S rRNA marker for pentastomid phylogenetics. Therefore, the aim of the present study was to determine the suitability and utility of 28S rRNA for differentiation of *Linguatula* spp.

## Materials and methods

Specimens of *Linguatula* spp. that have been morphologically identified in the Parasitology Laboratory at Charles Sturt University, Australia, were utilised for the genetic analyses of this study (Table 1). Morphological descriptions, including reference to deposited museum specimens, are available in Barton et al. (2020a), Barton et al. (2020b), Shamsi et al. (2020a) and Shamsi et al. (2020b) (see Online Resource 1).

**Table 1:** Details of sequences of species of *Linguatula* used in this study. Except for MN065508, all remaining 28S rRNA sequences were generated from this study. References listed for *Linguatula* *nuttalli* and *Linguatula* *serrata* are for 18S rRNA and *CoxI* sequences

ID	Species name	Locality	Host	Developmental stage	GenBank accession number			Reference
					28S	18 s	COI	
1	*L.* *nuttalli*	Africa	Buffalo	Nymph	OM304814	MN906667	MN905329	Shamsi et al. (2020b)
2	*L.* *nuttalli*	Africa	Buffalo	Nymph	OM304815	MN906673	MN905335	Shamsi et al. (2020b)
3	*L.* *nuttalli*	Africa	Buffalo	Nymph	OM304816	MN906674	MN905336	Shamsi et al. (2020b)
4	*L.* *nuttalli*	Africa	Buffalo	Nymph	OM304817	MN906672	MN905330	Shamsi et al. (2020b)
5	*L.* *nuttalli*	Africa	Buffalo	Nymph	OM304818	MN906675	MN905338	Shamsi et al. (2020b)
6	*L.* *nuttalli*	Africa	Buffalo	Nymph	OM304819	MN906670	MN905334	Shamsi et al. (2020b)
7	*L.* *nuttalli*	Africa	Lion	Adult female	OM304820	MN906671	MN905331	Shamsi et al. (2020b)
8	*L.* *nuttalli*	Africa	Lion	Adult	OM304821	MN906668	MN905332	Shamsi et al. (2020b)
9	*L.* *serrata*	Australia	Cow	Nymph	OM304822	MN889436	MN893765	Shamsi et al. (2020a)
10	*L.* *serrata*	Australia	Cow	Nymph	OM304823			
11	*L.* *serrata*	Australia	Cow	Nymph	OM304824			
12	*L.* *serrata*	Australia	Cow	Nymph	OM304825			
13	*L.* *serrata*	Australia	Dog	Adult female	OM304826			
14	*L.* *serrata*	Australia	Dog	Adult male	OM304827			
15	*L.* *serrata*	Australia	Dog	Adult female	OM304828	MN889438	MN893767	Shamsi et al. (2020a)
16	*L.* *serrata*	Australia	Dog	Adult male	OM304829			
17	*L.* *serrata*	Australia	Dog	Adult female	OM304830			
18	*L.* *serrata*	Australia	Dog	Adult male	OM304831	MN889440	MN893769	Shamsi et al. (2020a)
19	*L.* *serrata*	Australia	Fox	Adult male	OM304832	MN889437	MN893766	Shamsi et al. (2020a)
20	*L.* *serrata*	Australia	Fox	Adult female	OM304833	MN889439	MN893768	Shamsi et al. (2020a)
21	*L.* *serrata*	Australia	Fox	Adult male	OM304834			
22	*L.* *serrata*	Australia	Fox	Adult female	OM304835			
23	*L.* *serrata*	Australia	Fox	Adult male	OM304836			
24	*L.* *serrata*	Australia	Rabbit	Nymph	OM304837	MT196141	MT198822	Barton et al. (2020a, b)
25	*L.* *serrata*	Australia	Red-necked wallaby	Nymph	OM304838	MT367681	MT371890	Barton et al. (2020b)
26	*L.* *serrata*	Australia	Red-necked wallaby	Nymph	OM304839	MT367682	MT371891	Barton et al. (2020b)
27	*L.* *serrata*	Australia	Red-necked wallaby	Nymph	OM304840	MT367683	MT371892	Barton et al. (2020b)
28	*L.* *serrata*	Australia	Red-necked wallaby	Nymph	OM304841	MT367685	MT371894	Barton et al. (2020b)
29	*Armillifer* *agkistrodontis*	China	Snake	Adult		FJ607339	FJ607340	Chen et al. (2010)
30	*Levisunguis* *subaequalis*	USA	Mosquitofish	Nymph	MN065508			Woodyard et al. (2019a, b)

DNA extraction was performed using DNeasy Blood and Tissue Kits (Qiagen, Australia) according to the modified protocol of the manufacturer detailed in Shamsi et al. (2017a). A pair of primers were newly designed for the nuclear 28S rRNA region. The primer sequences are Ling_28SFm 5′ AGCTCATCGCCGAACCCT 3′ and Ling_28SRm 5′ATAGTTCACCATCTTTCGGGTCC 3′. PCR amplification was performed using GoTaq DNA polymerase (Promega, Australia) as per the manufacturer’s instructions. Cycling was initiated with a 2-min initial denaturation at 95 °C and followed by 40 cycles of denaturation at 95 °C for 30 s, annealing at 55 °C for 30 s and extension for 1 min at 72 °C. The cycle was concluded with a final extension at 72 °C for 10 min. PCR amplicons of 28S rRNA region were bidirectionally sequenced using the PCR primers by the Australian Genome Research Facility (Brisbane, Queensland, Australia). Sequences were deposited in GenBank with accession numbers OM304814-OM304821 (*L.* *nuttalli*) and OM304822-OM304841 (*L.* *serrata*).

*Cox1* and 18S rRNA sequences for the same specimens as used for the generation of the 28S rRNA gene sequences were obtained from GenBank (Table 1). All sequences were aligned with ClustalW in BioEdit (Hall 1999). Alignments were manually adjusted and truncated into 941, 1728 and 1256 bp for *Cox1*, 18S rRNA and 28S rRNA, respectively. Indels were ignored for analysis. Pairwise genetic distances among samples are shown as K2P genetic distance, and a number of differences were calculated by MEGA X (Kumar et al. 2018). Neighbour joining trees showing the grouping of species for the three genes were generated by the same software. Sequences from *Levisunguis* *subaequalis* (MN065508 for 28S rRNA) and *Armillifer* *agkistrodontis* (FJ607340 for *Cox1* and FJ607339 for 18S rRNA) were used as outgroups.

## Results and discussion

**Fig. 1:**
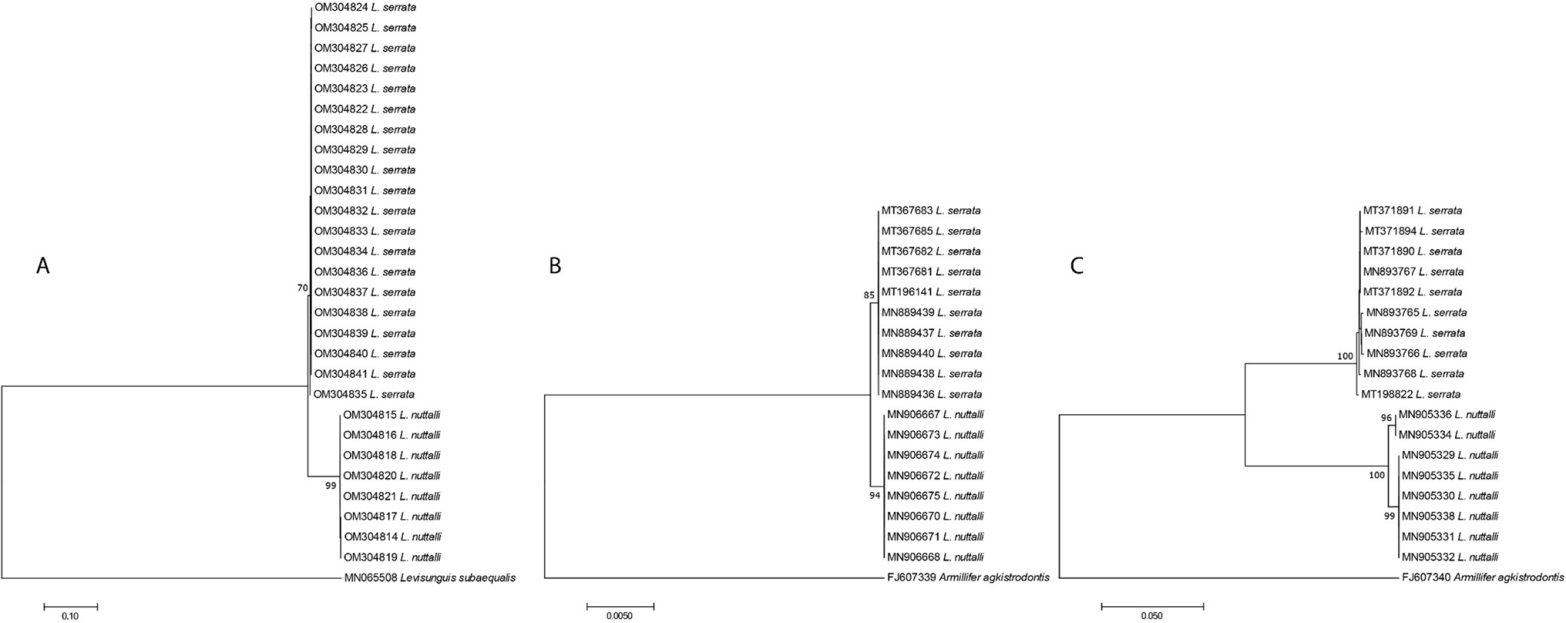
Neighbour joining tree showing the grouping of *Linguatula* *serrata* and *Linguatula* *nuttalli* for **A** 28S sequences, **B** 18S sequences and **C** *Cox1* sequences used in this study. All 28S sequences, except the outgroup species, were obtained from this research. Indels were ignored from analysis

This study presents 28S rRNA gene sequences for members of the genus *Linguatula*. Sequences of 28S rRNA were successfully obtained from samples of *L.* *serrata* (20 sequences: 9 collected from nymphs from various intermediate hosts and 11 collected from adults from wild dogs (*Canis* *familiaris*) and foxes (*Vulpes* *vulpes*); all collected in Australia) and *L.* *nuttalli* (8 sequences: 6 collected from nymphs from buffalo (*Syncerus* *caffer*) and 2 collected from adults from lions (*Panthera* *leo*); all collected in South Africa) (Fig. 1A; Table 1).

Across the three gene regions analysed, there was clear separation of *L.* *serrata* and *L.* *nuttalli* *with* 6.17–6.46% (68–70 out of 1256 base pairs) (Fig. 1A; Online Resource 2) and 13.09–13.31% (109–113 out of 941 base pairs) (Fig. 1C; Online Resource 4) interspecific difference for 28S rRNA and *Cox1*, respectively. This range of interspecific variation for *Cox1* was similar to that found in previous research with 9.8% difference between *L.* *serrata* and *L.* *arctica* (Gjerde 2013) and 12% between *L.* *serrata* and *L.* *recurvata* (Pérez-Flores et al. 2019). The results for 18S rRNA were much lower with 0.23% (4 out of 1728 base pairs) interspecific difference (Fig. 1B; Online Resource 3). This is consistent, however, with previous research, showing low levels of interspecific differences, with a 0.1% difference (2 of 1830 base pairs) between *L.* *serrata* and *L.* *arctica* (Gjerde 2013; Mohanta and Itagaki 2017). These results highlight that there may be multiple species of *Linguatula* in Iran, as suggested by Shamsi et al. (2020a), based on the results of Ghorashi et al. (2016) with interspecific differences within *L.* *serrata* sequences ranging from 0 to 2.9% for 18S rRNA.

Levels of intraspecific variation also differed between the three gene regions. Both *L.* *serrata* and *L.* *nuttalli* showed 0% intraspecific variation in the 18S sequences. However, *L.* *serrata* showed 0–0.26% and 0–0.43% intraspecific variation, while *L.* *nuttalli* showed 0–0.17% and 0–0.86% intraspecific variation at 28S and *Cox1*, respectively. Given the high level of differences between the two recognised species at both the 28S and *Cox1* genes, it is unlikely that this level of intraspecific variation is due to a species complex. However, more research needs to be undertaken, obtaining sequences from different life cycle stages and hosts across a wide geographical range, and for different species of *Linguatula*, to ensure all potential variabilities have been accounted for.

The intraspecific variation for 28S rRNA sequences for *L.* *serrata* was generally 0%, except for three sequences which showed consistent differences: two sequences obtained from nymphs collected from a cow (*Bos* *taurus*) (OM304824, OM304825) (0.17%) and an adult female collected from a fox (OM304835) (up to 0.26%). Sequences for *L.* *nuttalli* showed similar levels of intraspecific variation, with most having 0% difference, except for sequences obtained from three of the nymphal specimens. These nymphs showed 0.09% (OM304817, OM304819) and 0.17% (OM304814) difference to the other sequences (Online Resource 2).

Within *L.* *serrata*, most *Cox1* sequences varied by 0.11–0.21%, with a few sequences having no variation; the sequence obtained from the nymph collected from the rabbit (*Oryctolagus* *cuniculus*) (MT198822) showed 0.21–0.43% difference to all the other *L.* *serrata* sequences (Online Resource 4). Within *L.* *nuttalli*, most sequences had no variation except for two nymphs which consistently had 0.86% difference to the other sequences (MN905334, MN905336); one of these was the same nymph that showed variation in the 28S rRNA sequences. Intraspecific variation in *Cox1* sequences for *L.* *serrata* from various Iranian samples show lower values of differences. Despite finding a 9–10.9% difference between *L.* *serrata* and *L.* *arctica* sequences, Ghorashi et al. (2016) found only 0.4–3.1% differences within Iranian samples, and an overall diversity of 0.7–1.8% to the *L.* *serrata* sequence of Gjerde (2013). As with some of the sequences obtained in this study, Ghorashi et al. (2016) noted differences in sequences between samples collected from different host animals. Sudan et al. (2018) also found intra-specific variability in the sequences of *L.* *serrata*, with different groupings of samples: one group showed nymphs from Indian buffaloes with a nymph from a Bangladeshi cow and an Iranian sheep (from Kerman); another group were all collected from the Kerman region of Iran, from cattle, sheep and goats; the last group was a mix of sequences from specimens collected from various hosts and geographical locations, including Peru, Bangladesh and Iran. External to these groups were nymphs collected from sheep from Tabriz, Iran (KF830143 & KF830144; Ghorashi et al., 2016), and the *L.* *serrata* adult from the dog in Norway (Gjerde 2013). However, Sudan et al. (2018) reported that there was only a 0–0.2% difference between the sequences. Bootstrap support for all the trees created in this study was confident (> 70%). The *Cox1* tree had higher support than the 28S and 18S trees, probably due to the higher number of fixed mutations. The clade of *L.* *serrata* in the 28S tree had slightly lower bootstrap support, probably due to a few unique point mutations in sequences OM304824, OM304825 and OM304835, as discussed above.

As has been shown in this study, there are substantially different levels of intra and inter-specific variability between sequences across the three different markers examined. However, the levels of variability appear to match results found for other pentastome genera. For example, intraspecific variability for nymphs of *Sebekia* *mississippiensis* was reported as 0–0.09% for 28S and 0–1.03% for *Cox1* (Woodyard et al. 2019a, b) and < 1% for *Alofia* *merki* for *Cox1* (J. Morgan, pers. comm.), whereas there were no differences within *Reighardia* *sternae* for 18S rRNA (Literák et al. 2017). Thus, it appears that there are low levels of intraspecific variability within the three genetic markers across many pentastomid genera. Interspecific differences for the genus *Sebekia* (as presented in Barton and Morgan (2016)) were > 5% for 28 s rRNA and > 14% for *Cox1* but < 0.5% for 18S rRNA (J. Morgan, pers. comm.). These results, as well as the results reported in this study, show that 28S rRNA and *Cox1* have higher levels of interspecific variability, showing potentially better species-level differentiation compared to the results for 18S rRNA.

Woodyard et al. (2019b) stressed that host records that do not provide adequate morphological or molecular data to justify independent specific diagnoses must be regarded cautiously. In earlier studies, we have provided comprehensive morphological measurements in combination with molecular characterisation. More research is required to sample specimens of *Linguatula* from as wide a host and geographical range as possible, utilising a combination of morphological and molecular characterisation to ultimately determine the levels of intraspecific variability across the various molecular markers.

## Supplementary Information

Below is the link to the electronic supplementary material.

Supplementary file1 (DOCX 21 KB)

Supplementary file2 (DOCX 27 KB)

Supplementary file3 (DOCX 20 KB)

Supplementary file4 (DOCX 20 KB)

## Data Availability

All sequences generated in this study have been submitted to GenBank.
