# Supplementary material for: 28S rRNA sequences for Linguatula spp
Source: Parasitol Res. 2022 Apr 1;121(6):1799–804. doi: 10.1007/s00436-022-07507-6 (PMC9098581; doi:10.1007/s00436-022-07507-6)
Supplement: Supplementary file 1 — Supplementary file1 (DOCX 21 KB) [file 436_2022_7507_MOESM1_ESM.docx]

**Online Resource 1.** Comparison of measurements of specimens of *Linguatula serrata* and *Linguatula nuttalli*. Hook measurements follow templates provided in Shamsi at al. (2020a) and Barton et al. (2020a). Measurements are in microns unless otherwise stated. Data is presented as a mean with range in parentheses.

|  | *Linguatula serrata* | | | | | | *Linguatula nuttalli* | | |
| --- | --- | --- | --- | --- | --- | --- | --- | --- | --- |
| Stage | Nymph | Nymph | Nymph | Nymph | Adult | Adult | Nymph | Nymph | Adult |
| Sex | Male | Female | Male | Female | Male | Female | Female | Male | Female |
| No. specimens | 4 | 2 | 3 | 8 | 6 | 6 | 4 | 1 | 1 |
| Total Length (mm) | 5.01 (3.83-5.48) | 4.44 (4.13-4.75) | 3.63 (3.5-3.9) | 4.34 (3.70-5.60) | 16.4 (15-18) | 59.9 (48-70) | 6.2 (4-9.8) | 4.9 (-) | 47.0 |
| Max Width (mm) | 1.16 (1.03-1.23) | 0.96 (0.93-1.00) | 0.90 (0.76-1.2) | 0.96 (0.72-1.2) | 3.2 (2.5-4) | 7.9 (7-8.5) | 1.35 (1.0-1.7) | 1.7 (-) | 6.0 |
| No. annuli | 87 (83-90) | 86 (83-88) | 81 (75-86) | 88 (83-92) | 80 (75-89) | 92 (86-109) | 123 (110-145) | >100 | 128 |
| Anterior Hook |  |  |  |  |  |  |  |  |  |
| AC | 152.5 (150-155) | 170 (155-180) | 160.2 (141-177) | 170 (-) | 129.6 (113-150) | 160.6 (145-175) | 240 (-) | - | 480 |
| AD | 185 (-) | 210 (200-220) | 196.5 (193-202) | 200 (-) | 211 (195-235) | 252.5 (235-280) | 335 (-) | - | 800 |
| BC | 140 (130-150) | 144.2 (133-155) | 124.2 (90-140) | 146.5 (140-153) | 101.7 (90-115) | 142.5 (105-165) | 220 (-) | - | 400 |
| CD | 83.3 (80-90) | 96.7 (85-95) | 77.3 (66-93) | 72.5 (70-75) | 100.8 (75-130) | 132.5 (105-165) | 170 (-) | - | 480 |
| AB | 80 (75-85) | 91.7 (85-95) | 89 (84-102) | 103 (-) | 82.6 (75-100) | 92 (75-105) | 160 (-) | - | 250 |
| DAP | 115 (-) | 109.2 (103-115) | - | 139 (128-150) | N/A | N/A | - | - | N/A |
| FL | 322.5 (318-330) | 326.7 (310-335) | 327.3 (304-368) | 323.9 (175-388) | 265 | 510 (500-520) | 500 (-) | - | - |
| Posterior Hook |  |  |  |  |  |  |  |  |  |
| AC | 165 (-) | 160 (155-165) | 148.3 (131-168) | 172.2 (160-176) | 136 (120-150) | 150 (140-160) | 265 (-) | - | 520 |
| AD | 210 (-) | 197.5 (190-205) | 187 (183-191) | 201.2 (188-210) | 212 (200-230) | 270 (250-190) | 360 (-) | - | 870 |
| BC | 141.3 (140-143) | 140 (130-150) | 112.3 (99-132) | 140 (131-155) | 108.3 (100-120) | 145 (110-160) | 230 (-) | - | 450 |
| CD | 85 (-) | 102.5 (90-115) | 89 (72-106) | 80.2 (56-96) | 103.3 (90-130) | 145 (110-180) | 140 (-) | - | 520 |
| AB | 92.5 (-) | 87.3 (82-93) | 89 (80-95) | 89.2 (81-100) | 94 (80-130) | 100 (90-110) | 180 (-) | - | 280 |
| DAP | 113.8 (110-118) | 110 (-) | - | 129.3 (118-150) | N/A | N/A | 200 (-) | - | N/A |
| FL | 326.3 (320-333) | 337.5 (335-340) | 330 (316-357) | 341.4 (257-377) | 145 (130-160) | 470 | 510 (-) | - | - |
| Buccal Cadre L | 195 (190-200) | 205 (-) | 142 (120-166) | 163 (153-190) | 180 | 227.5 (130-300) |  |  |  |
| Buccal Cadre W | 105 (-) | 95 (-) | 61.7 (60-63) | 89.2 (63-125) | 180 | 197.5 (90-270) |  |  |  |
| Copulatory Spicule L |  |  |  |  | 448.5 (430-460) |  |  |  |  |
| Copulatory Spicule W |  |  |  |  | 162.5 (140-175) | - |  |  |  |
| Host species | *Notamacropus rufogriseus* | | *Oryctolagus cuniculus* | | *Canis familiaris* | | *Syncerus caffer* | | *Panthera leo* |
| Geographical location | Australia | | Australia | | Australia | | South Africa | | South Africa |
| Reference | Barton et al. (2020b) | | Barton et al. (2020a) | | Shamsi et al. (2020a) | | Shamsi et al. (2020b) | | Shamsi et al. (2020b) |

**References:**

Barton DP, Baker A, Porter M, Zhu X, Jenkins DJ, Shamsi S (2020a) Verification of rabbits as intermediate hosts for *Linguatula serrata* (Pentastomida) in Australia. Parasitology Research 119:1552-1562

Barton DP, Porter M, Baker A, Zhu X, Jenkins D, Shamsi S (2020b) First report of nymphs of the introduced pentastomid, *Linguatula serrata*, in red-necked wallabies (*Notamacropus rufogriseus*) in Australia. Australian Journal of Zoology 67:106-113

Shamsi S, Barton DP, Zhu X, Jenkins DJ (2020a) Characterisation of the tongue worm, *Linguatula serrata* (Pentastomida: Linguatulidae), in Australia. International Journal for Parasitology: Parasites and Wildlife 11:149-157 doi:https://doi.org/10.1016/j.ijppaw.2020.01.010

Shamsi S, et al. (2020b) Occurrence and characterisation of tongue worms, *Linguatula* spp., in South Africa. International Journal for Parasitology: Parasites and Wildlife 11:268-281 doi:10.1016/j.ijppaw.2020.03.002
