# Supplementary material for: 28S rRNA sequences for Linguatula spp
Source: Parasitol Res. 2022 Apr 1;121(6):1799–804. doi: 10.1007/s00436-022-07507-6 (PMC9098581; doi:10.1007/s00436-022-07507-6)
Supplement: Supplementary file 2 — Supplementary file2 (DOCX 27 KB) [file 436_2022_7507_MOESM2_ESM.docx]

**Online Resource 2.** Pairwise genetic distance matrix of the 28S regions of sequences used in this study, shown as K2P genetic distance (above the diagonal) and number of differences (below the diagonal). Indels were deleted from analysis. Please refer to Table 1 in the Manuscript for the ID of the sequences.

|  | 1 | 2 | 3 | 4 | 5 | 6 | 7 | 8 | 9 | 10 | 11 | 12 | 13 | 14 | 15 | 16 | 17 | 18 | 19 | 20 | 21 | 22 | 23 | 24 | 25 | 26 | 27 | 28 | 30 |
| --- | --- | --- | --- | --- | --- | --- | --- | --- | --- | --- | --- | --- | --- | --- | --- | --- | --- | --- | --- | --- | --- | --- | --- | --- | --- | --- | --- | --- | --- |
| 1 |  | 0.17% | 0.17% | 0.09% | 0.17% | 0.09% | 0.17% | 0.17% | 6.36% | 6.36% | 6.46% | 6.46% | 6.36% | 6.36% | 6.36% | 6.36% | 6.36% | 6.36% | 6.36% | 6.36% | 6.36% | 6.26% | 6.36% | 6.36% | 6.36% | 6.36% | 6.36% | 6.36% | 53.07% |
| 2 | 2 |  | 0.00% | 0.09% | 0.00% | 0.09% | 0.00% | 0.00% | 6.17% | 6.17% | 6.27% | 6.27% | 6.17% | 6.17% | 6.17% | 6.17% | 6.17% | 6.17% | 6.17% | 6.17% | 6.17% | 6.26% | 6.17% | 6.17% | 6.17% | 6.17% | 6.17% | 6.17% | 52.70% |
| 3 | 2 | 0 |  | 0.09% | 0.00% | 0.09% | 0.00% | 0.00% | 6.17% | 6.17% | 6.27% | 6.27% | 6.17% | 6.17% | 6.17% | 6.17% | 6.17% | 6.17% | 6.17% | 6.17% | 6.17% | 6.26% | 6.17% | 6.17% | 6.17% | 6.17% | 6.17% | 6.17% | 52.70% |
| 4 | 1 | 1 | 1 |  | 0.09% | 0.00% | 0.09% | 0.09% | 6.26% | 6.26% | 6.36% | 6.36% | 6.26% | 6.26% | 6.26% | 6.26% | 6.26% | 6.26% | 6.26% | 6.26% | 6.26% | 6.36% | 6.26% | 6.26% | 6.26% | 6.26% | 6.26% | 6.26% | 52.87% |
| 5 | 2 | 0 | 0 | 1 |  | 0.09% | 0.00% | 0.00% | 6.17% | 6.17% | 6.27% | 6.27% | 6.17% | 6.17% | 6.17% | 6.17% | 6.17% | 6.17% | 6.17% | 6.17% | 6.17% | 6.26% | 6.17% | 6.17% | 6.17% | 6.17% | 6.17% | 6.17% | 52.70% |
| 6 | 1 | 1 | 1 | 0 | 1 |  | 0.09% | 0.09% | 6.26% | 6.26% | 6.36% | 6.36% | 6.26% | 6.26% | 6.26% | 6.26% | 6.26% | 6.26% | 6.26% | 6.26% | 6.26% | 6.36% | 6.26% | 6.26% | 6.26% | 6.26% | 6.26% | 6.26% | 52.87% |
| 7 | 2 | 0 | 0 | 1 | 0 | 1 |  | 0.00% | 6.17% | 6.17% | 6.27% | 6.27% | 6.17% | 6.17% | 6.17% | 6.17% | 6.17% | 6.17% | 6.17% | 6.17% | 6.17% | 6.26% | 6.17% | 6.17% | 6.17% | 6.17% | 6.17% | 6.17% | 52.70% |
| 8 | 2 | 0 | 0 | 1 | 0 | 1 | 0 |  | 6.17% | 6.17% | 6.27% | 6.27% | 6.17% | 6.17% | 6.17% | 6.17% | 6.17% | 6.17% | 6.17% | 6.17% | 6.17% | 6.26% | 6.17% | 6.17% | 6.17% | 6.17% | 6.17% | 6.17% | 52.70% |
| 9 | 70 | 68 | 68 | 69 | 68 | 69 | 68 | 68 |  | 0.00% | 0.09% | 0.09% | 0.00% | 0.00% | 0.00% | 0.00% | 0.00% | 0.00% | 0.00% | 0.00% | 0.00% | 0.17% | 0.00% | 0.00% | 0.00% | 0.00% | 0.00% | 0.00% | 51.92% |
| 10 | 70 | 68 | 68 | 69 | 68 | 69 | 68 | 68 | 0 |  | 0.09% | 0.09% | 0.00% | 0.00% | 0.00% | 0.00% | 0.00% | 0.00% | 0.00% | 0.00% | 0.00% | 0.17% | 0.00% | 0.00% | 0.00% | 0.00% | 0.00% | 0.00% | 51.92% |
| 11 | 71 | 69 | 69 | 70 | 69 | 70 | 69 | 69 | 1 | 1 |  | 0.00% | 0.09% | 0.09% | 0.09% | 0.09% | 0.09% | 0.09% | 0.09% | 0.09% | 0.09% | 0.26% | 0.09% | 0.09% | 0.09% | 0.09% | 0.09% | 0.09% | 52.12% |
| 12 | 71 | 69 | 69 | 70 | 69 | 70 | 69 | 69 | 1 | 1 | 0 |  | 0.09% | 0.09% | 0.09% | 0.09% | 0.09% | 0.09% | 0.09% | 0.09% | 0.09% | 0.26% | 0.09% | 0.09% | 0.09% | 0.09% | 0.09% | 0.09% | 52.12% |
| 13 | 70 | 68 | 68 | 69 | 68 | 69 | 68 | 68 | 0 | 0 | 1 | 1 |  | 0.00% | 0.00% | 0.00% | 0.00% | 0.00% | 0.00% | 0.00% | 0.00% | 0.17% | 0.00% | 0.00% | 0.00% | 0.00% | 0.00% | 0.00% | 51.92% |
| 14 | 70 | 68 | 68 | 69 | 68 | 69 | 68 | 68 | 0 | 0 | 1 | 1 | 0 |  | 0.00% | 0.00% | 0.00% | 0.00% | 0.00% | 0.00% | 0.00% | 0.17% | 0.00% | 0.00% | 0.00% | 0.00% | 0.00% | 0.00% | 51.92% |
| 15 | 70 | 68 | 68 | 69 | 68 | 69 | 68 | 68 | 0 | 0 | 1 | 1 | 0 | 0 |  | 0.00% | 0.00% | 0.00% | 0.00% | 0.00% | 0.00% | 0.17% | 0.00% | 0.00% | 0.00% | 0.00% | 0.00% | 0.00% | 51.92% |
| 16 | 70 | 68 | 68 | 69 | 68 | 69 | 68 | 68 | 0 | 0 | 1 | 1 | 0 | 0 | 0 |  | 0.00% | 0.00% | 0.00% | 0.00% | 0.00% | 0.17% | 0.00% | 0.00% | 0.00% | 0.00% | 0.00% | 0.00% | 51.92% |
| 17 | 70 | 68 | 68 | 69 | 68 | 69 | 68 | 68 | 0 | 0 | 1 | 1 | 0 | 0 | 0 | 0 |  | 0.00% | 0.00% | 0.00% | 0.00% | 0.17% | 0.00% | 0.00% | 0.00% | 0.00% | 0.00% | 0.00% | 51.92% |
| 18 | 70 | 68 | 68 | 69 | 68 | 69 | 68 | 68 | 0 | 0 | 1 | 1 | 0 | 0 | 0 | 0 | 0 |  | 0.00% | 0.00% | 0.00% | 0.17% | 0.00% | 0.00% | 0.00% | 0.00% | 0.00% | 0.00% | 51.92% |
| 19 | 70 | 68 | 68 | 69 | 68 | 69 | 68 | 68 | 0 | 0 | 1 | 1 | 0 | 0 | 0 | 0 | 0 | 0 |  | 0.00% | 0.00% | 0.17% | 0.00% | 0.00% | 0.00% | 0.00% | 0.00% | 0.00% | 51.92% |
| 20 | 70 | 68 | 68 | 69 | 68 | 69 | 68 | 68 | 0 | 0 | 1 | 1 | 0 | 0 | 0 | 0 | 0 | 0 | 0 |  | 0.00% | 0.17% | 0.00% | 0.00% | 0.00% | 0.00% | 0.00% | 0.00% | 51.92% |
| 21 | 70 | 68 | 68 | 69 | 68 | 69 | 68 | 68 | 0 | 0 | 1 | 1 | 0 | 0 | 0 | 0 | 0 | 0 | 0 | 0 |  | 0.17% | 0.00% | 0.00% | 0.00% | 0.00% | 0.00% | 0.00% | 51.92% |
| 22 | 69 | 69 | 69 | 70 | 69 | 70 | 69 | 69 | 2 | 2 | 3 | 3 | 2 | 2 | 2 | 2 | 2 | 2 | 2 | 2 | 2 |  | 0.17% | 0.17% | 0.17% | 0.17% | 0.17% | 0.17% | 51.95% |
| 23 | 70 | 68 | 68 | 69 | 68 | 69 | 68 | 68 | 0 | 0 | 1 | 1 | 0 | 0 | 0 | 0 | 0 | 0 | 0 | 0 | 0 | 2 |  | 0.00% | 0.00% | 0.00% | 0.00% | 0.00% | 51.92% |
| 24 | 70 | 68 | 68 | 69 | 68 | 69 | 68 | 68 | 0 | 0 | 1 | 1 | 0 | 0 | 0 | 0 | 0 | 0 | 0 | 0 | 0 | 2 | 0 |  | 0.00% | 0.00% | 0.00% | 0.00% | 51.92% |
| 25 | 70 | 68 | 68 | 69 | 68 | 69 | 68 | 68 | 0 | 0 | 1 | 1 | 0 | 0 | 0 | 0 | 0 | 0 | 0 | 0 | 0 | 2 | 0 | 0 |  | 0.00% | 0.00% | 0.00% | 51.92% |
| 26 | 70 | 68 | 68 | 69 | 68 | 69 | 68 | 68 | 0 | 0 | 1 | 1 | 0 | 0 | 0 | 0 | 0 | 0 | 0 | 0 | 0 | 2 | 0 | 0 | 0 |  | 0.00% | 0.00% | 51.92% |
| 27 | 70 | 68 | 68 | 69 | 68 | 69 | 68 | 68 | 0 | 0 | 1 | 1 | 0 | 0 | 0 | 0 | 0 | 0 | 0 | 0 | 0 | 2 | 0 | 0 | 0 | 0 |  | 0.00% | 51.92% |
| 28 | 70 | 68 | 68 | 69 | 68 | 69 | 68 | 68 | 0 | 0 | 1 | 1 | 0 | 0 | 0 | 0 | 0 | 0 | 0 | 0 | 0 | 2 | 0 | 0 | 0 | 0 | 0 |  | 51.92% |
| 30 | 433 | 431 | 431 | 432 | 431 | 432 | 431 | 431 | 426 | 426 | 427 | 427 | 426 | 426 | 426 | 426 | 426 | 426 | 426 | 426 | 426 | 426 | 426 | 426 | 426 | 426 | 426 | 426 |  |
