# Supplementary material for: 28S rRNA sequences for Linguatula spp
Source: Parasitol Res. 2022 Apr 1;121(6):1799–804. doi: 10.1007/s00436-022-07507-6 (PMC9098581; doi:10.1007/s00436-022-07507-6)
Supplement: Supplementary file 4 — Supplementary file4 (DOCX 20 KB) [file 436_2022_7507_MOESM4_ESM.docx]

**Online Resource 4.** Pairwise genetic distance matrix of the *Cox1* regions of sequences used in this study, shown as K2P genetic distance (above the diagonal) and number of differences (below the diagonal). Indels were deleted from analysis. Please refer to Table 1 in the manuscript for the ID of the sequences.

|  | 1 | 2 | 3 | 4 | 5 | 6 | 7 | 8 | 9 | 15 | 18 | 19 | 20 | 24 | 25 | 26 | 27 | 28 | 29 |
| --- | --- | --- | --- | --- | --- | --- | --- | --- | --- | --- | --- | --- | --- | --- | --- | --- | --- | --- | --- |
| 1 |  | 0.00% | 0.86% | 0.00% | 0.00% | 0.86% | 0.00% | 0.00% | 13.22% | 13.22% | 13.36% | 13.22% | 13.22% | 12.95% | 13.22% | 13.22% | 13.22% | 13.36% | 33.46% |
| 2 | 0 |  | 0.86% | 0.00% | 0.00% | 0.86% | 0.00% | 0.00% | 13.22% | 13.22% | 13.36% | 13.22% | 13.22% | 12.95% | 13.22% | 13.22% | 13.22% | 13.36% | 33.46% |
| 3 | 8 | 8 |  | 0.86% | 0.86% | 0.00% | 0.86% | 0.86% | 13.09% | 13.09% | 13.22% | 13.09% | 13.09% | 12.82% | 13.09% | 13.09% | 13.09% | 13.22% | 33.11% |
| 4 | 0 | 0 | 8 |  | 0.00% | 0.86% | 0.00% | 0.00% | 13.22% | 13.22% | 13.36% | 13.22% | 13.22% | 12.95% | 13.22% | 13.22% | 13.22% | 13.36% | 33.46% |
| 5 | 0 | 0 | 8 | 0 |  | 0.86% | 0.00% | 0.00% | 13.22% | 13.22% | 13.36% | 13.22% | 13.22% | 12.95% | 13.22% | 13.22% | 13.22% | 13.36% | 33.46% |
| 6 | 8 | 8 | 0 | 8 | 8 |  | 0.86% | 0.86% | 13.09% | 13.09% | 13.22% | 13.09% | 13.09% | 12.82% | 13.09% | 13.09% | 13.09% | 13.22% | 33.11% |
| 7 | 0 | 0 | 8 | 0 | 0 | 8 |  | 0.00% | 13.22% | 13.22% | 13.36% | 13.22% | 13.22% | 12.95% | 13.22% | 13.22% | 13.22% | 13.36% | 33.46% |
| 8 | 0 | 0 | 8 | 0 | 0 | 8 | 0 |  | 13.22% | 13.22% | 13.36% | 13.22% | 13.22% | 12.95% | 13.22% | 13.22% | 13.22% | 13.36% | 33.46% |
| 9 | 112 | 112 | 111 | 112 | 112 | 111 | 112 | 112 |  | 0.21% | 0.11% | 0.21% | 0.32% | 0.43% | 0.21% | 0.21% | 0.21% | 0.32% | 31.33% |
| 15 | 112 | 112 | 111 | 112 | 112 | 111 | 112 | 112 | 2 |  | 0.11% | 0.21% | 0.11% | 0.21% | 0.00% | 0.00% | 0.00% | 0.11% | 31.50% |
| 18 | 113 | 113 | 112 | 113 | 113 | 112 | 113 | 113 | 1 | 1 |  | 0.11% | 0.21% | 0.32% | 0.11% | 0.11% | 0.11% | 0.21% | 31.50% |
| 19 | 112 | 112 | 111 | 112 | 112 | 111 | 112 | 112 | 2 | 2 | 1 |  | 0.32% | 0.43% | 0.21% | 0.21% | 0.21% | 0.32% | 31.67% |
| 20 | 112 | 112 | 111 | 112 | 112 | 111 | 112 | 112 | 3 | 1 | 2 | 3 |  | 0.32% | 0.11% | 0.11% | 0.11% | 0.21% | 31.33% |
| 24 | 110 | 110 | 109 | 110 | 110 | 109 | 110 | 110 | 4 | 2 | 3 | 4 | 3 |  | 0.21% | 0.21% | 0.21% | 0.32% | 31.50% |
| 25 | 112 | 112 | 111 | 112 | 112 | 111 | 112 | 112 | 2 | 0 | 1 | 2 | 1 | 2 |  | 0.00% | 0.00% | 0.11% | 31.50% |
| 26 | 112 | 112 | 111 | 112 | 112 | 111 | 112 | 112 | 2 | 0 | 1 | 2 | 1 | 2 | 0 |  | 0.00% | 0.11% | 31.50% |
| 27 | 112 | 112 | 111 | 112 | 112 | 111 | 112 | 112 | 2 | 0 | 1 | 2 | 1 | 2 | 0 | 0 |  | 0.11% | 31.50% |
| 28 | 113 | 113 | 112 | 113 | 113 | 112 | 113 | 113 | 3 | 1 | 2 | 3 | 2 | 3 | 1 | 1 | 1 |  | 31.67% |
| 29 | 252 | 252 | 250 | 252 | 252 | 250 | 252 | 252 | 239 | 240 | 240 | 241 | 239 | 240 | 240 | 240 | 240 | 241 |  |
